# Colonisation potential of the bark beetle ( *Taphrorychus bicolor* ) on beech logs and logging residues: ecological context and implications for pest management in forests

**DOI:** 10.1002/ps.70741

**Published:** 2026-03-30

**Authors:** Ivana Henzlová, Karolina Resnerová, Jiří Trombik, Juraj Galko, Jaroslav Holuša

**Affiliations:** ^1^ Faculty of Forestry and Wood Sciences Czech University of Life Sciences Suchdol Czech Republic; ^2^ National Forest Centre Zvolen Slovak Republic

**Keywords:** colonisation success, emergence traps, entry holes, *Fagus sylvatica*, forest pest management, reproductive success, *Taphrorychus bicolor*

## Abstract

**BACKGROUND:**

The bark beetle *Taphrorychus bicolor* has been traditionally classified as a secondary pest of European beech. However, climate‐induced stress and increased host availability may enhance its importance in forest ecosystems. Despite the high abundance of this pest, data on its reproductive biology and colonisation preferences remain limited. Therefore, we conducted three experiments, each addressing a different aspect of *T. bicolor* ecology (colonisation density, population–substrate relationships, and reproductive success), to assess its colonisation behaviour on beech logs and logging residues at selected Central European sites.

**RESULTS:**

In the first experiment, both trap catches and entry hole densities declined between years, but no significant relationship between trap catches and colonisation density was detected after accounting for year effects. In the second experiment, reproductive success was quantified by comparing the numbers of entry and exit holes and emerged adults from colonised logging residues. Exit holes were moderately correlated with beetle emergence, whereas entry holes showed weaker predictive power. This pattern may reflect mortality or re‐entry behaviour. In the third experiment, *T. bicolor* was the dominant coloniser across five study sites, whereas ambrosia beetles were sporadic, suggesting limited interspecific competition under the conditions studied. Colonisation density was significantly reduced under full sun exposure but unaffected by pheromone‐ and kairomone‐based lures or log dimensions.

**CONCLUSIONS:**

*T. bicolor* showed consistent reproductive success on small‐diameter beech logging residues with limited interspecific competition. Exit holes provide a reliable proxy for emergence, supporting logging residue management. © 2026 The Author(s). *Pest Management Science* published by John Wiley & Sons Ltd on behalf of Society of Chemical Industry.

## Graphical abstract

Beech residues left in shaded or semi‐shaded conditions pose a substantial risk of local *Taphrorychus bicolor* population outbreaks. The rapid removal or placement of residues in sun‐exposed locations can help mitigate this risk.

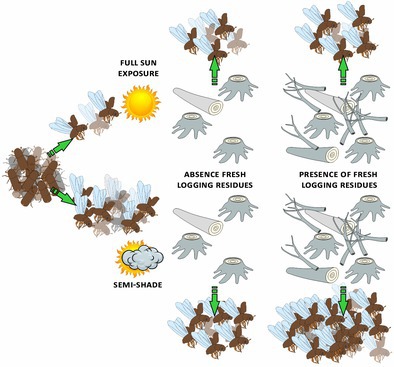

## 1 INTRODUCTION

Bark beetles (Coleoptera: Curculionidae: Scolytinae) are among the most ecologically and economically important insect groups associated with forest ecosystems. Although their impacts have been well documented in coniferous forests, increasing evidence indicates that broadleaved forests are also affected, particularly under prolonged drought conditions intensified by climate change.^1^, ^2^ Despite the high diversity of bark beetles associated with deciduous trees, most are secondary pests that typically colonise weakened or stressed hosts.^3^, ^4^

In Europe, climate‐driven changes are altering host–pest interactions by weakening tree defence mechanisms through rising temperatures and extended drought periods.^5^, ^6^ Although coniferous trees remain the primary focus of bark beetle research, deciduous species, particularly European beech (*Fagus sylvatica* L.), also merit attention. This species is the most common deciduous tree in the Czech Republic, accounting for approximately 11.9% of the total tree composition in Europe.^7^ It dominates mesic, acidic forest habitats at elevations ranging from 300 to 900 m a.s.l.^8^, ^9^

The retention of deadwood in forest ecosystems is a key factor supporting the biodiversity of saproxylic insects.^10^ However, under Central European forestry conditions, the amount of deadwood is often limited owing to concerns about the proliferation of potentially harmful species and associated economic risks.^11^ This creates a management trade‐off between biodiversity conservation and forest protection, particularly under changing climatic conditions that may facilitate shifts in the behaviour of secondary pest species.

Among the bark beetle species colonising European beech, *Taphrorychus bicolor* (Herbst, 1793) has traditionally been classified as a secondary pest. It typically infests stressed or dying trees and may accelerate tree mortality under drought conditions.^12^, ^13^ Nevertheless, increasing population densities of *T. bicolor* have been linked to notable ecological and economic impacts.^14^, ^15^ Recent studies have suggested that *T. bicolor* may also pose a risk to seemingly healthy beech trees under adverse environmental conditions.^16^, ^17^ Colonisation of living tissues can trigger host defence reactions, resulting in necrotic bark lesions that are frequently accompanied by secondary bacterial or fungal infections and have been described as a specific bark disease.^18^ These lesion‐type injuries can substantially reduce timber quality, thereby increasing the economic relevance of the species.^15^ Importantly, unlike aggressive bark beetle species, *T. bicolor* does not exhibit coordinated mass‐attack behaviour, and increased population densities therefore do not necessarily translate into widespread tree mortality.

*T. bicolor* primarily develops in beech trees (*Fagus sylvatica*, *F. orientalis* Lipsky), but has also been reported from oaks (*Quercus robur* L., *Q. dalechampii* Ten., *Q. polycarpa* Schur., *Q. cerris* L., *Q. pedunculata* Ehrh.), hornbeam (*Carpinus betulus* L.), birch (*Betula spp*.), goat willow (*Salix caprea* L.), and hazel (*Corylus avellana* L.).^15^, ^19^, ^20^ The host tree is initially colonised primarily by males, which construct mating chambers beneath the bark and begin releasing aggregation pheromones that attract females and additional males.^12^ As *T. bicolor* is a polygamous species, a single male typically attracts three to eight females to a mating chamber. The main components of the aggregation pheromone are (±)‐bicolorin and acetophenone.^21^, ^22^ After mating, each female excavates a maternal gallery and deposits eggs along its entire length,^23^ from which larval galleries subsequently develop. Depending on seasonal conditions, the development of one generation lasts approximately 8–12 weeks; however, if larvae or pupae overwinter within the frass, development may extend to more than 8 months.^24^ In Central Europe, *T. bicolor* typically produces two generations per year.^20^, ^24^ Flight activity of the parental generation occurred from late April to June, followed by a filial generation from late June to August.^15^, ^24^, ^25^

In addition to *T. bicolor*, several ambrosia beetles, such as *Trypodendron domesticum* (Linnaeus, 1758) and *Xylosandrus germanus* (Blandford, 1894), have co‐occurred on beech logs and logging residues. *T. domesticum* has been commonly detected in stem traps,^26^, ^27^ whereas the invasive *X. germanus* has been observed at higher abundances than *T. bicolor* in some regions.^28^, ^29^ Although bark beetles and ambrosia beetles exploit different tissues of the same host—phloem and xylem, respectively—indirect competition may occur, mediated by substrate condition or colonisation timing. Most existing studies on *T. bicolor* have focused on the number of entry holes in standing trees^19^ or logging residues.^30^, ^31^ However, data on the reproductive success, colonisation dynamics, and potential interaction of *T. bicolor* with co‐occurring saproxylic species remain limited. The spatiotemporal overlap of these beetle guilds in disturbed forest habitats raises questions about possible interference, particularly given the pest status of some ambrosia beetles under changing climatic conditions. In some cases, behavioural interference may also result from chemical interactions, for example certain kairomonal blends can suppress the responsiveness of ambrosia beetles.^32^, ^33^

No outbreaks of *T. bicolor* have been reported in the Czech Republic.^25^, ^31^ Nevertheless, increasing availability of suitable breeding material, such as logging residues, combined with climate‐induced stress of beech stands, may enhance the ecological relevance of this species. Addressing existing knowledge gaps on its colonisation preferences, interactions with co‐occurring species and reproductive success is therefore essential for evidence‐based forest pest management, particularly with respect to the handling and spatial placement of logging residues in beech‐dominated stands.

Under Central European forestry conditions, management of beech forests increasingly involves decisions on whether, where, and how long logging residues and freshly cut wood should be retained in the stand. Such material represents an important substrate for saproxylic biodiversity but may simultaneously serve as breeding material for bark beetles. Understanding which substrates are colonised by *T. bicolor*, under which microclimatic conditions, and how reproduction proceeds on logging residues is therefore directly relevant for forest pest management.

Accordingly, in this study, we aimed to examine (i) whether locally recorded pheromone trap catches co‐vary with colonisation density of logging residues under operational forest conditions and identify potential limitations of trap‐based monitoring at small spatial and temporal scales, (ii) whether beech logging residues provide suitable conditions for successful reproduction of *T. bicolor*, and (iii) how light conditions and the presence of lures targeting other bark beetle guilds affect colonisation of beech logs by *T. bicolor*.

## 2 MATERIAL AND METHODS

### 2.1 Experiment 1: Local co‐variation between pheromone trap catches and colonisation of beech logging residues

**Figure 1:**
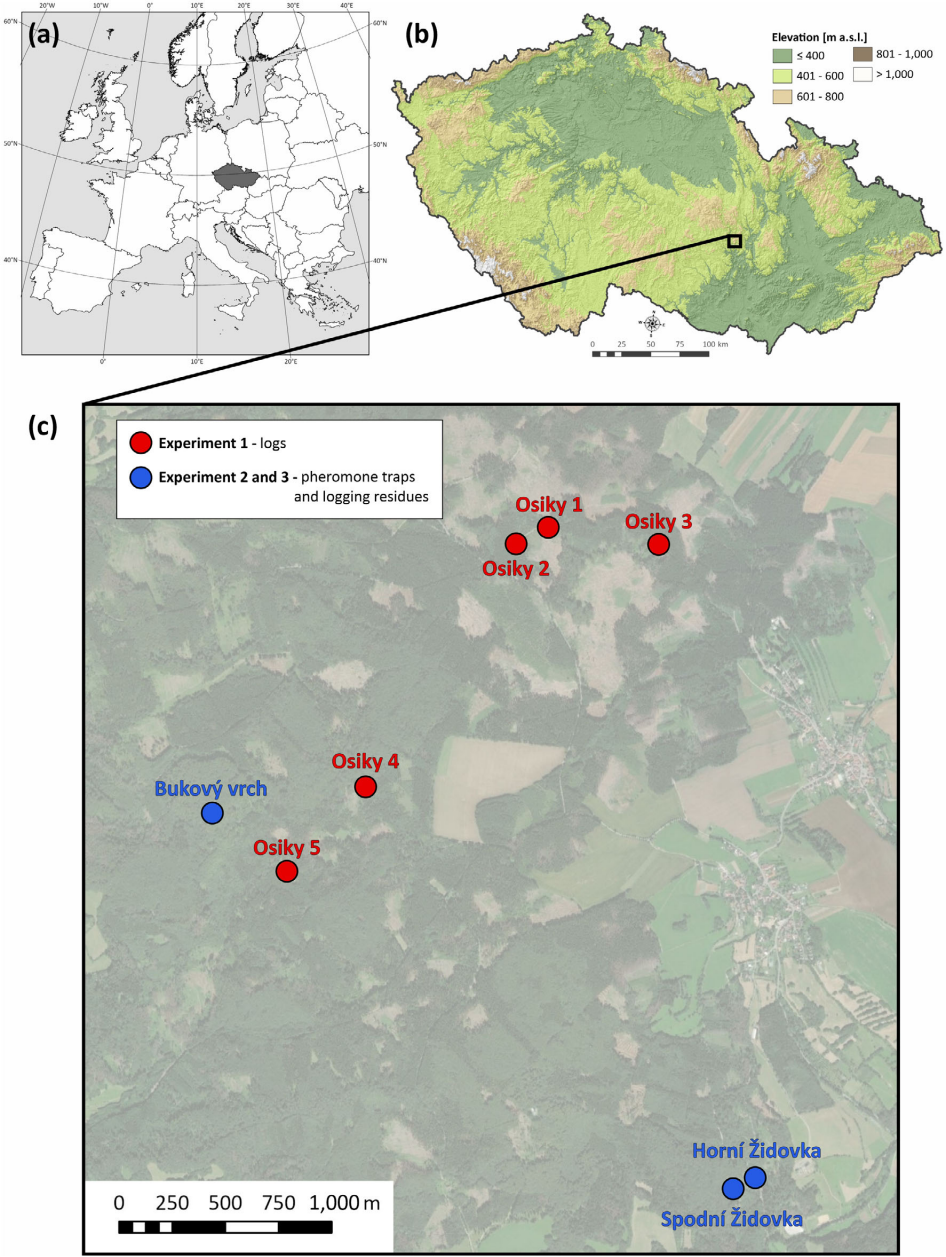
Study sites during the experiments in 2022 and 2024 in the Czech Republic.

Data were collected in 2022 and 2024 from three forest stands in the Czech Republic: Horní Židovka (49.44386 N, 16.42239 E), Spodní Židovka (49.44363 N, 16.42152 E), and Bukový vrch (49.45690 N, 16.38521 E) (Fig. 1). Logging residue piles were placed in forest stands dominated by *F. sylvatica*, comprising at least 90% of the canopy, extending over an area of at least 1 ha with an average stand age of 100 years, and situated at approximately 600 m a.s.l.

**Figure 2:**
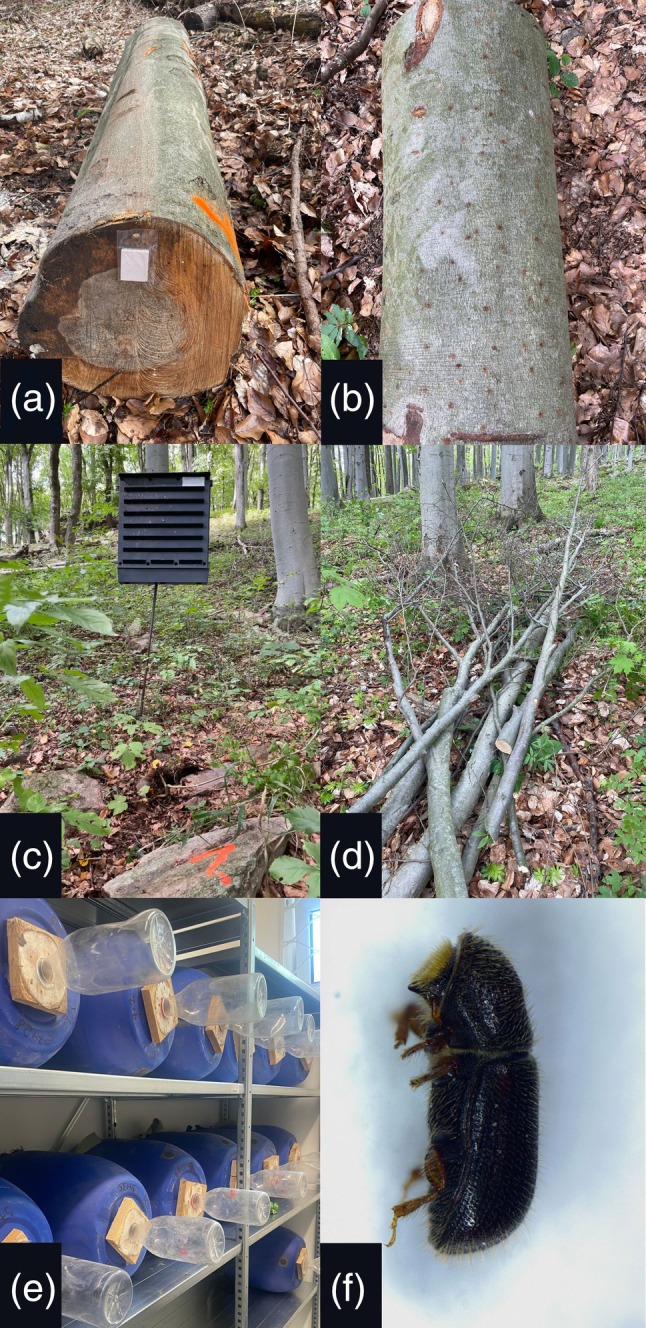
Overview of the experimental setups. (A) Beech logs (*Fagus sylvatica*) baited with lineatin, (B) detail of logs showing beetle entry holes, (C) Theysohn‐type pheromone trap, (D) pile of beech branches, (E) emergence traps with beech branches, and (F) female *Taphrorychus bicolor*.

In 2022, three logging‐residue piles were installed per pheromone trap, whereas in 2024, only one pile was installed per trap (Fig. 2(C),(D)). Logging residue piles were placed within the stand before the onset of flight activity in early March. Within a 50‐m radius of each pile, a Theysohn‐type pheromone trap was installed in a semi‐shaded location. In early April, each trap was baited with a pheromone dispenser containing the species‐specific lure Beech Bark Beetle Lure (AlphaScents, Canby, OR, USA), which releases the pheromone compound bicolorin.^34^ Trap catches were summed from the lure deployment to the census date (20 June).

Each installed pile comprised 15 pieces of logging residue, averaging 158 cm (length) × 5 cm (diameter) in 2022 and 179 × 5 cm in 2024. At the end of June, following a decline in flight activity, the piles were thoroughly examined. Colonisation density was calculated separately for each individual logging residue piece (branch) by counting the number of entry holes and measuring their dimensions; the values were then converted to standardised surface area densities (entry holes per 1 m^2^) using the calculated lateral surface area of a cylinder.

This experiment was not designed to calibrate pheromone trap catches against absolute population density. Rather, it represents a pragmatic field setup reflecting common operational monitoring, characterised by limited spatial extent, short temporal coverage, and unavoidable methodological adjustments between years.

### 2.2 Experiment 2: Evaluation of the reproductive success of *T. bicolor* on logging residues

In June 2024, 25 colonised logging residues from Experiment 1 (3 study sites) were randomly selected and transported to the laboratory. For each logging residue, the number of entry holes, logging residue length, and mean diameter at the midpoint were recorded. The mean length of the selected logging residues was 2.0 ± 0.4 m, with a mean diameter of 2.8 ± 0.9 cm. Logging residues were cut into 40‐cm sections and placed in plastic emergence traps (Fig. 2(E)) under constant room conditions: 21 ± 2 °C, 40–60% relative humidity, and a 16:8 h light:dark photoperiod. Entry holes were recorded at the time of installation of the logging residues into the emergence traps on 20 June, i.e. before the start of emergence monitoring. According to our records, the first adults began to leave the branches on 24 June 2024. Emergence traps were inspected weekly, and emerging adults were removed and counted. After adult emergence declined and no individuals emerged for at least 2 consecutive weeks, all logging residues were removed from the emergence traps. Before debarking, we recorded the final total number of bark perforations (holes) on each section; exit holes were calculated as (final perforations) − (entry holes recorded at installation). After debarking, non‐target species were identified and the remaining bark beetle adults beneath the bark were recorded (Fig. 2(F)). All counts were expressed per m^2^.

### 2.3 Experiment 3: Colonisation of beech logs by *T. bicolor* in relation to kairomonal treatments and microclimate conditions

Five study sites were selected near the village of Osiky (49.4551378°N, 16.4199636°E), located northwest of Brno (Czech Republic; Fig. 1). The study sites were situated near managed forest stands with a dominant (approximately 80%) representation of beech (*Fagus sylvatica*), an average age of 100 years, and an altitude of approximately 600 m a.s.l. The stands are located on moderately deep modal cambisols typical of the Bohemian‐Moravian Highlands and corresponding to common beech site conditions in the region. The study period coincided with a dry and thermally above‐average growing season, indicating that the trees were likely exposed to seasonal drought stress at the time of colonisation.

One landing yard used for storing harvested broadleaved timber was selected at each site. In March, before the start of *T. bicolor* and ambrosia beetle flight activity, one triplet of beech logs was placed in a transect with randomised treatments. This triplet originated from healthy, freshly harvested beech trees whose age and growth characteristics (diameter and height) were comparable to those of approximately 100‐year‐old trees growing in nearby stands. The logs had an average length of 205 ± 4.3 cm and a diameter of 30 ± 3.2 cm. The logs were spaced at regular intervals of over 15 m, with each study site being 500–1000 m apart.

To assess whether the presence of kairomonal and pheromone lures targeting ambrosia beetles influences the colonisation patterns of *T. bicolor*, and to evaluate the potential for indirect interspecific interactions, we applied three experimental treatments. In the first treatment, logs were baited with the kairomone Xylowit® (Witasek PflanzenSchutz GmbH, Feldkirchen in Kärnten, Austria), designed to attract *Xylosandrus* spp. This lure consists of two separately packed transparent pouches (A and B), deployed together. Pouch A contains α‐pinene (95%) and pouch B contains ethanol (95%).

The second treatment involved the application of Lineatin Kombi® (Witasek PflanzenSchutz GmbH), a pheromone blend targeting *Trypodendron* spp. This aggregation lure includes lineatin (3,3,7‐trimethyl‐2,9‐dioxatricyclo‐[3.3.1^0 4,7]nonane), guaiacol (2‐methoxyphenol), nonyl aldehyde, and 3‐hydroxy‐2‐methyl‐2‐butanone.

The third treatment consisted of unbaited control logs, with no attractant applied. One log per treatment was installed at each of five study sites, resulting in a total of 15 logs (*n* = 5 per treatment). The logs were placed at standardised distances and exposed simultaneously under similar microclimatic conditions.

Each log was inspected for bark beetles by examining entry holes, the presence of adults, and frass at regular weekly intervals after installation (Fig. 2(B)). Light exposure was classified in the field using a standardised protocol: shade = ≤1 h of direct solar incidence between 10:00 and 15:00 local time with visual canopy cover ≥75%, semi‐shade = 1–3 h and 40–75%, and sun = ≥3 h and <40%; classifications were assigned independently by two observers at installation and verified during the first two weekly checks with *N* = 6, *N* = 4 and *N* = 5 logs per category, respectively. After recording the colonisation of *T. bicolor*, the density of each species on the logs was recorded on 20 June 2024. Subsequently, the number of infestations of each species per log area was recalculated, and the colonisation density of bark beetles on beech logs was assessed.

### 2.4 Statistical analysis

All analyses were performed in R version 4.3.2^35^ using the packages ggplot2,^36^ MASS,^37^ car,^38^ and emmeans.^39^

Entry‐hole density (no. m^−2^) was analysed using negative binomial generalised linear models (GLMs) with a log link function. Fixed effects included the natural logarithm of trap catches [log(trap catches)], site (three levels; reference = Bukový vrch), and year (2022 *vs* 2024). Results are reported as incidence rate ratios (IRR = exp(β)) with 95% confidence intervals. Model diagnostics included checks for overdispersion, residual plots, and Cook's distance. Model selection was based on Akaike's Information Criterion (AIC).

Relationships between colonisation density, emergence (exit holes), and trap catches were analysed using simple linear regression. Bark surface area (m^2^) was used as a covariate to standardise emergence and colonisation rates across samples of varying size. The association between entry and exit holes was also examined to assess the link between colonisation density and reproductive output. For each model, the regression slope, Pearson's correlation coefficient (*r*), coefficient of determination (*R* ^2^), and *P* value were reported. The seasonal dynamics of *T. bicolor* were presented as boxplots of raw counts (number of individuals per period), without logarithmic transformation.

Entry‐hole density was modelled using negative binomial GLMs with an identity link, allowing for interpretation of additive effects in original units (holes per m^2^). Fixed effects included light conditions (shade, semi‐shade, full sun), treatment (control, lineatin, α‐pinene, ethanol), and log diameter. Results are reported as adjusted mean differences with 95% confidence intervals, *z*‐statistics, and *P* values.

For predictors with more than two levels, *post hoc* pairwise comparisons were conducted using estimated marginal means (EMMs) with Tukey's correction for multiple testing, based on the fitted model.

## 3 RESULTS

### 3.1 Experiment 1: Local co‐variation between pheromone trap catches and colonisation of beech logging residues

Over the 2 sampling years (2022 and 2024), 127 043 individuals of *T. bicolor* were trapped, which corresponded to a mean catch of 12 375 ± 7146 individuals per trap in 2022 and 5223 ± 1818 in 2024. The average density of entry holes per m^2^ of logging residues also declined from 425 ± 313 in 2022 to 39 ± 25 in 2024.

**Figure 3:**
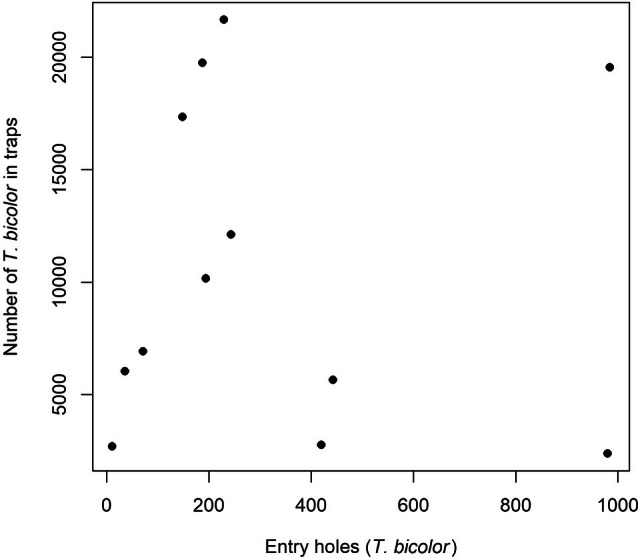
Scatterplot showing the relationship between the number of *Taphrorychus bicolor* entry holes on logging residues and the number of beetles captured in pheromone traps.

Across the studied sites and years, no significant association was observed between pheromone trap catches and entry‐hole density on logging residues in either raw (Fig. 3) or log‐transformed models (adjusted *R* ^2^ < 0.07). Including site and year as covariates increased the explained variance (adjusted *R* ^2^ = 0.565), with year being the only significant factor (*P* = 0.0055), indicating substantially lower infestation in 2024 than in 2022. The Poisson model indicated significance of all predictors; however, diagnostic tests revealed extreme overdispersion (dispersion = 190.4), indicating substantial violation of model assumptions. The negative binomial model substantially improved the model fit (AIC = 160.7, residual deviance = 12.76 on 7 df). The only significant predictor was year (*β* = −1.156, *P* < 0.001), with significantly lower infestation in 2024 than in 2022 (Table 1). Within the spatiotemporal scope of this study, neither pheromone trap catches nor study site explained variation in entry‐hole density.

**Table 1:** Negative binomial GLM (log link)

Variable	Estimate	Standard error	*z* value	*Pr*(>|*z*|)
(Intercept)	2343.686	454.495	5.157	2.51e‐07 ***
Log traps	−0.079	0.276	−0.285	0.776
Site Horní Židovka	−0.619	0.458	−1.351	0.177
Site Spodní Židovka	0.025	0.433	0.058	0.954
Year	−1.156	0.224	−5.154	2.54e‐07 ***

Results shown as IRR (exp(β)) with 95% CI, z, and p. Reference levels: site = Bukový vrch; year = 2022. AIC = 160.66. Number of Fisher scoring iterations was 1. *** indicates significance at *P* < 0.001.

### 3.2 Experiment 2: Evaluation of the reproductive success of *T. bicolor* on logging residues

The density of *T. bicolor* entry holes across 25 logging residues averaged 92.3 ± 50.3 holes per m^2^. Other Scolytinae species were detected only sporadically; *X. germanus* was found on six residues and *T. domesticum* on eight residues, each represented by a single gallery system. No additional bark‐ or wood‐boring species were recorded during the study period.

**Figure 4:**
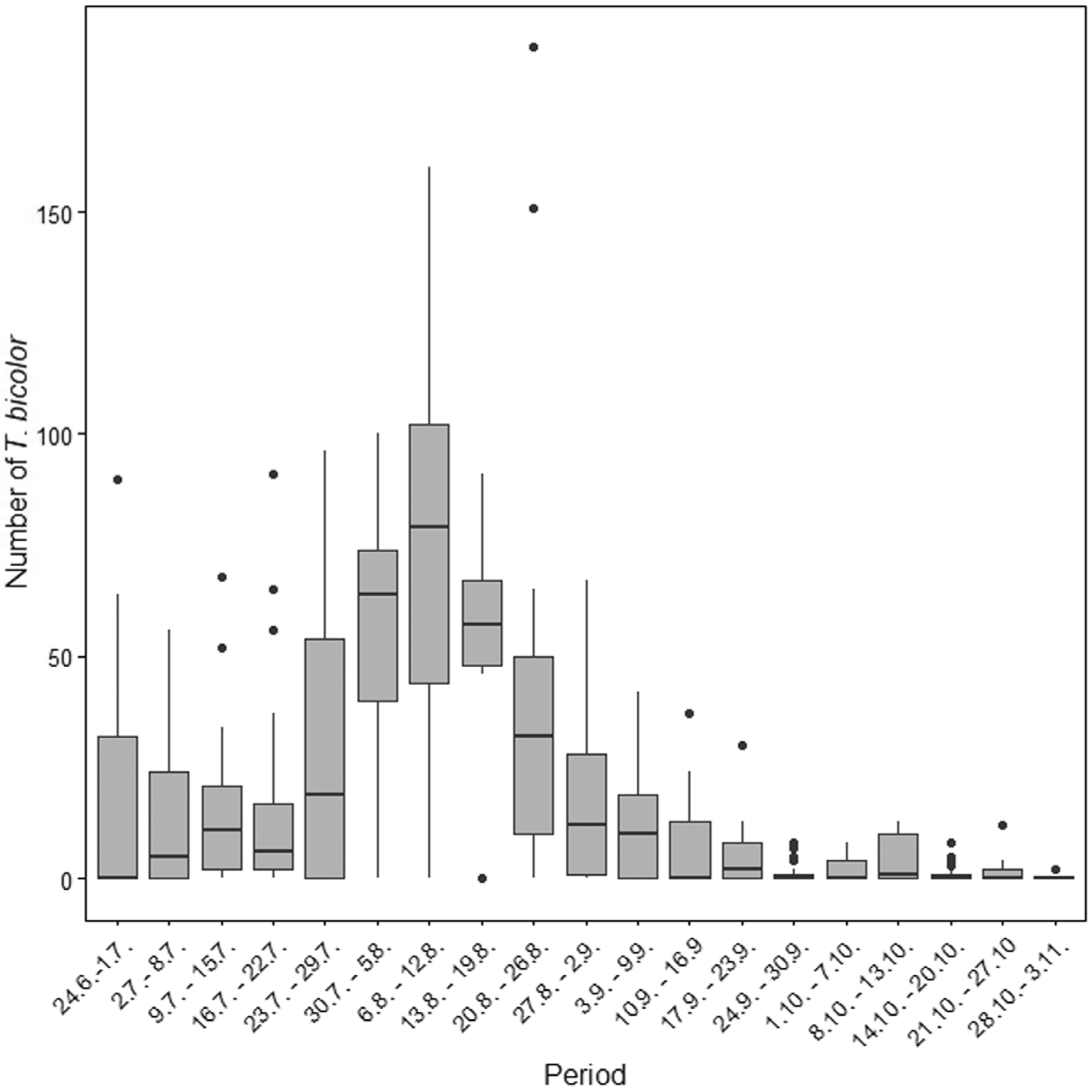
Flight activity of *Taphrorychus bicolor* (number of individuals per period) based on samples from emergence traps collected from late June to early November in 2024.

The emergence of *T. bicolor* adults commenced immediately following the installation of residues in the emergence traps in June and continued uninterrupted until early November, with peak activity observed in late July and early August (Fig. 4).

On average, 943.7 ± 751.3 exit holes per m^2^ were recorded (range 12.7–2657.0, median 719.5). Based on the number of exit holes, reproductive success was estimated at 10.8 offspring per gallery system. By contrast, the number of emerging individuals captured in the emergence traps was consistently lower than the number of recorded exit holes, with a mean density of 503.1 ± 428.8 individuals per m^2^, which corresponded to a realised production of 6.1 offspring per gallery system. Although the number of emerging individuals recorded from the traps was lower, the number of exit holes provides a more reliable estimate of reproductive performance, as it reflects the total number of beetles that successfully completed development in the host material. Therefore, reproductive success was evaluated based on the number of exit holes in the subsequent analysis.

**Figure 5:**
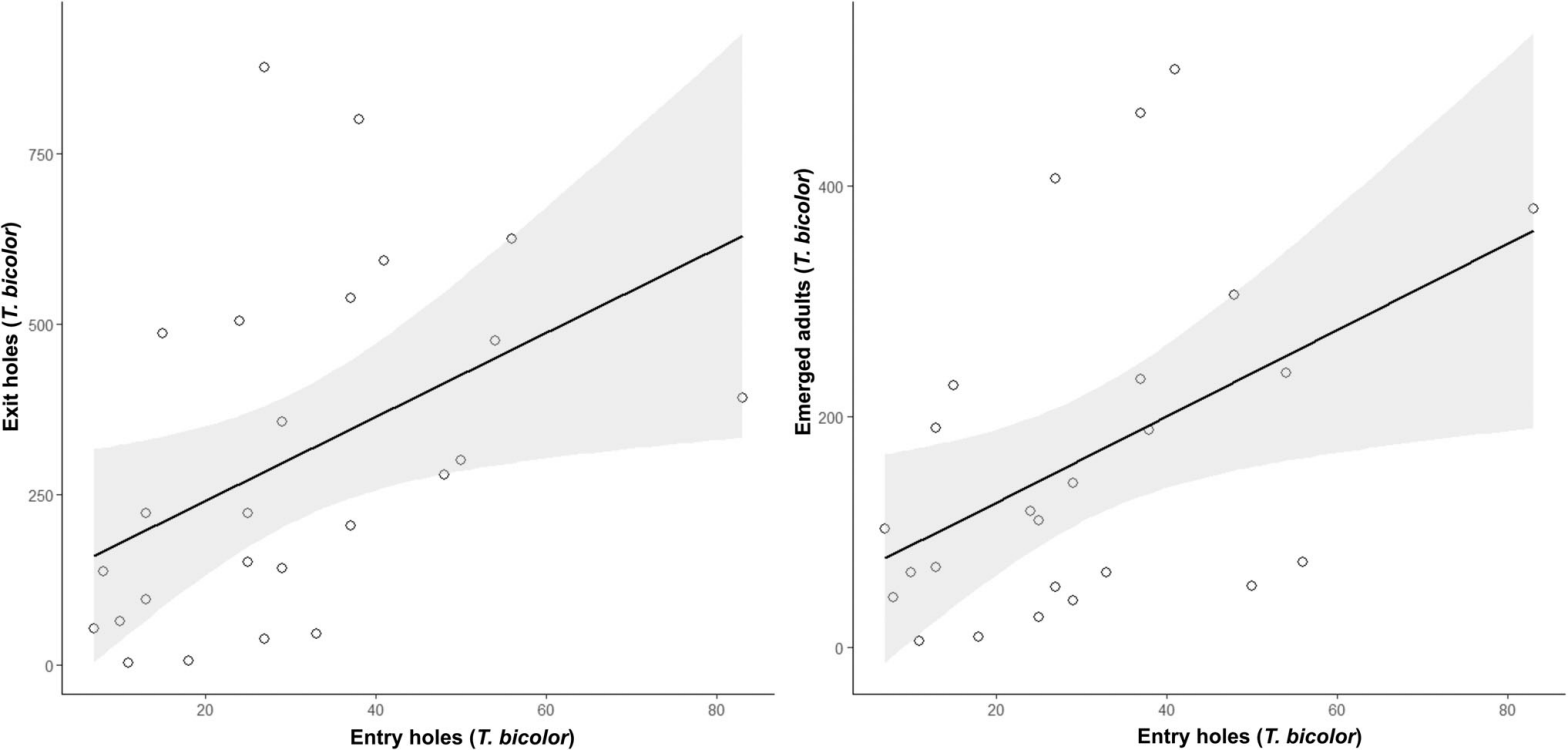
Relationships between the numbers of entry holes, exit holes (left), and emerged adults (right) of *Taphrorychus bicolor* in infested logging residues in 2024.

The surface area of the logging residues had no effect on either the number of exit holes or the ratio of F1 beetles per exit hole, with both relationships being weak and statistically non‐significant (*y* = 185.63 + 327.28*x*, *r* = 0.209, *P* > 0.05, *r* ^2^ = 0.044 and *y* = 0.47 + 0.36x, *r* = 0.132, *P* > 0.05, *r* ^2^ = 0.018, respectively). By contrast, significantly positive associations were found between colonisation density and beetle emergence. The number of exit holes increased with the number of entry holes (*y* = 117.56 + 6.16*x*, *r* = 0.446, *P* < 0.05, *r* ^2^ = 0.199), indicating that residues with more initial galleries tended to produce more exit holes, although the explanatory power of the model was low. The number of beetles emerging was moderately related to the number of exit holes (*y* = 53.75 + 0.36*x*, *r* = 0.622, *P* < 0.001, *r* ^2^ = 0.387). A significantly positive relationship was also observed between entry holes and beetle emergence (*y* = 50.94 + 3.74*x*, *r* = 0.464, *P* < 0.05, *r* ^2^ = 0.215; Fig. 5), suggesting that a higher colonisation density was generally associated with a greater number of emerging adults.

### 3.3 Experiment 3: Colonisation of beech logs by *T. bicolor* in relation to kairomonal treatments and microclimate conditions

At the five study sites in 2024, we investigated the colonisation density of *T. bicolor* and the occurrence of ambrosia beetles (*X. germanus* and *T. domesticum*) in response to treatment type and light conditions (Table 2). The dominant species across all treatments was *T. bicolor*, with densities ranging from 10.6 to 79.8 entry holes per m^2^. By contrast, *X. germanus* and *T. domesticum* were only sporadically present. *T. domesticum* was recorded at only one site on a log without a lure, whereas *X. germanus* appeared at three sites with low entry densities. Notably, the simultaneous occurrence of multiple species on the same log was recorded only in a single case, and the presence of ambrosia beetles was marginal. Given the lack of observable effects on gallery establishment or brood development, interspecific competition could not be inferred from our data. In addition to scolytine beetles, the fan‐bearing wood borer *Ptilinus pectinicornis* (Linnaeus, 1758) (Ptinidae) was detected exclusively on bark‐free log ends and was not associated with bark beetle galleries.

**Table 2:** Mean density (m^2^) of *Taphrorychus bicolor*, *Xylosandrus germanus*, and *Trypodendron domesticum* under different treatment types and light conditions across five study sites in 2024, with corresponding mean trap‐log length and diameter

Treatment	Light condition	Length (cm)	Diameter (cm)	*T. bicolor* (m^2^)	*X. germanus* (m^2^)	*T. domesticum* (m^2^)
Control	Shade	202.80	34.95	49.91	1.21	3.62
Control	Semi‐shade	206.50	34.90	50.35	–	–
Control	Sun	210.75	37.00	34.28	3.03	–
Lineatin	Shade	205.30	37.60	60.19	23.39	–
Lineatin	Semi‐shade	205.90	39.10	28.47	–	–
Lineatin	Sun	205.50	34.25	33.97	–	–
α‐pinene and ethanol	Shade	207.15	34.90	31.04	–	–
α‐pinene and ethanol	Semi‐shade	205.35	37.00	54.78	–	–
α‐pinene and ethanol	Sun	195.40	39.00	10.86	–	–

**Figure 6:**
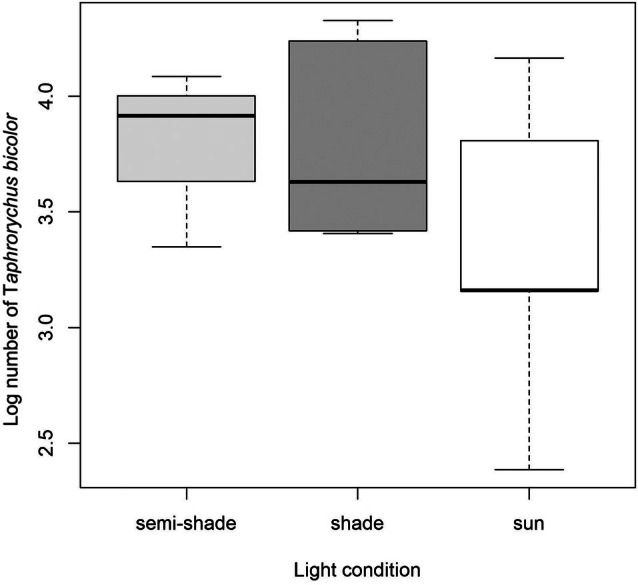
*Taphrorychus bicolor* entry‐hole density (log) by light conditions of beech logs at study sites in 2024 (for the definition of light conditions, see Material and methods).

The negative binomial regression model revealed that light conditions significantly affected beetle activity. Specifically, exposure to full sun significantly reduced *T. bicolor* colonisation compared with the semi‐shade reference category (*P* = 0.007). Logs exposed to shade showed a non‐significant decrease in beetle density compared with semi‐shaded logs (*P* = 0.289; Fig. 6).

Among the treatments, logs baited with α‐pinene and ethanol exhibited a trend toward reduced *T. bicolor* infestation compared with the control logs without a lure, although the effect was only marginally significant (*P* = 0.089). Lineatin Kombi did not significantly affect infestation levels (*P* = 0.451). Neither diameter was a statistically significant predictor (*P* = 0.595), suggesting that within the studied range, log size had minimal influence on colonisation.

*Post hoc* comparisons revealed that *T. bicolor* colonisation was significantly lower on logs exposed to full sun compared to semi‐shaded logs (*P* = 0.028). The difference between shade and sun was marginally non‐significant (*P* = 0.050), while shade and semi‐shade did not differ significantly (*P* = 0.857). No significant differences were found among treatments (*P* > 0.27 for all pairwise comparisons).

## 4 DISCUSSION

Our study provides new insights into the ecological preferences and reproductive dynamics of *T. bicolor* under field conditions. This discussion is structured according to the relative importance of the findings for forest pest management. First, we found that full sun exposure was associated with a significantly lower colonisation density compared with semi‐shaded conditions, suggesting that extreme light exposure may create unfavourable microclimatic conditions for successful reproduction. Second, despite this variation in colonisation, we observed high reproductive output across suitable substrates, indicating the potential for rapid population development under favourable conditions. Finally, no direct relationship was found between trap catches and colonisation density, highlighting the importance of interpreting flight activity data with caution when assessing population density in the field.

Among the factors examined, microclimatic conditions associated with light exposure emerged as the most consistent driver of colonisation success. Colonisation density was significantly lower under full sun exposure than under semi‐shaded conditions, indicating that extreme microclimatic conditions may limit successful development. Although the observed densities fall within the range previously reported for sun‐exposed beech material,^19^, ^30^ they contrast with studies documenting higher oviposition and exit‐hole densities of *T. bicolor* on sun‐exposed logging residues and branches.^40^ This discrepancy suggests that the response of *T. bicolor* to light conditions may be context‐dependent and influenced by interactions with other environmental factors. Taken together, our results suggest that extreme sun exposure, rather than shading *per se*, may impose the strongest constraints on successful colonisation of beech residues by *T. bicolor*, likely through microclimatic effects on wood moisture and temperature.^41^ In addition, prolonged sun exposure may promote bark cracking or partial detachment and generate temperatures exceeding the tolerance thresholds of the developing brood, further constraining successful development. Of note, however, the experiment was not originally designed to test the effect of solar exposure, and sample sizes across exposure categories were relatively small and unbalanced; therefore, the observed differences should be interpreted as indicative rather than as a definitive test of sun‐exposure effects.

Beyond colonisation patterns, the reproductive performance of *T. bicolor* on logging residues indicates a high potential for local population increase. Freshly cut beech residues proved to be highly suitable breeding substrates, with colonisation densities substantially exceeding those reported for entire trees.^15^ Exit‐hole densities and emergence data suggest that a single gallery system may give rise to multiple offspring, consistent with previously reported fecundity estimates and gallery structure in this species.^15^, ^23^

Although the number of emerging individuals was lower than the number of exit holes, exit holes provide a robust proxy for reproductive output because they directly reflect successful completion of development. The relatively modest strength of correlations between entry holes, exit holes, and emergence likely reflects a combination of generation overlap, recolonisation or microclimatic variability within the substrate, rather than methodological artefacts. Taken together, these results confirm that beech logging residues can support efficient reproduction of *T. bicolor* and thus represent an important driver of local population build‐up when suitable material is available.

The absence of a significant effect of logging‐residue size on colonisation density and reproductive success suggests that host quality rather than substrate dimensions governs the successful development of *T. bicolor*.^14^ The physiological condition of the wood, including moisture content and chemical properties, therefore appears to be more important for successful colonisation than morphometric characteristics such as surface area or volume. Similar conclusions have been drawn for other species, where host quality determines developmental success, whereas size only has a limited effect.^42^, ^43^ These findings support the interpretation that freshly cut beech residues provide favourable conditions for *T. bicolor* primarily due to their physiological state rather than their size.

Despite frequent co‐occurrence of other saproxylic beetles in beech stands, interspecific interactions appeared to play a minor role under the conditions studied. The very limited occurrence of ambrosia beetles, such as *X. germanus* and *T. domesticum*, even in the presence of kairomonal lures, suggests either a low local abundance or limited overlap in colonisation niches with *T. bicolor*. Although the absence of species‐specific positive controls constrains detailed inference, the observed patterns suggest that competitive interactions with other early‐colonising saproxylic beetles were unlikely to substantially influence the colonisation success or reproductive output of *T. bicolor* in the studied system.

Previous studies have indicated that *T. bicolor* commonly colonises beech material without artificial attraction. Spontaneous attacks on unbaited beech trees have been documented,^19^ and colonisation of logging residues and trap trees without pheromone use has been repeatedly reported.^15^ Moreover, the aggregation pheromone bicolorin has been described primarily as a monitoring tool rather than a prerequisite for host colonisation.^25^ In this context, we did not include a pheromone treatment for *T. bicolor*, as its presence was reliably expected under natural conditions.

In contrast to the clear effects of substrate and microclimate, pheromone trap catches showed limited explanatory power for colonisation density at the local scale examined. No consistent relationship was detected between trap catches and the density of colonisation in logging residues, indicating that the numbers of caught beetles recorded by pheromone traps do not necessarily reflect local infestation pressure under such conditions.

At the study site, *T. bicolor* completed two generations per year, as indicated by the bimodal seasonal activity pattern observed in pheromone trap catches and supported by previous observations from the same region. While this voltinism explains the prolonged flight activity recorded during the vegetation season, it does not imply that trap catches provide a reliable quantitative measure of local population size or reproductive output. This finding does not contradict those of previous studies demonstrating significant relationships between pheromone trap catches and subsequent infestation levels at broader spatial scales. Rather, it highlights the strong scale dependency of trap‐based monitoring. Robust correlations between trap catches and infestation levels have primarily been documented at landscape scales and under standardised monitoring designs,^44^, ^45^ whereas such relationships may be weak or obscured at local scales involving a limited number of traps and closely spaced sites.

At local spatial scales, pheromone trap catches are strongly influenced by methodological and environmental factors, including pheromone release rates, ambient temperature, and local trap position, which can introduce substantial variability independent of true population density.^46^, ^47^ Accordingly, while pheromone traps remain valuable tools for monitoring flight phenology and relative activity patterns, their use as quantitative proxies for local population size or infestation pressure should be approached with caution.

## 5 CONCLUSIONS

The beech bark beetle *T. bicolor* was the dominant coloniser of beech logs. Colonisation success was markedly reduced under full sun exposure, highlighting the importance of microclimatic conditions. At the local spatial scale examined, pheromone trap catches did not reliably reflect colonisation density in logging residues and should therefore be interpreted with caution when used as stand‐level indicators. Freshly cut beech residues and branches proved to be highly suitable breeding substrates, with several adults developing per gallery system, confirming the high reproductive potential of this species. From a forest management perspective, residues left in semi‐shaded conditions can pose a substantial risk of local population increase. The rapid removal or relocation of residues in sun‐exposed locations can help to mitigate this risk.

## CONFLICT OF INTEREST

The authors declare that they have no conflict of interest.

## Data Availability

The data that support the findings of this study are available from the corresponding author upon reasonable request.
